# Triggers and alleviating factors for fatigue in Parkinson’s disease

**DOI:** 10.1371/journal.pone.0245285

**Published:** 2021-02-04

**Authors:** Iris Lin, Briana Edison, Sneha Mantri, Steven Albert, Margaret Daeschler, Catherine Kopil, Connie Marras, Lana M. Chahine

**Affiliations:** 1 Department of Neurology, University of Pittsburgh, Pittsburgh, PA, United States of America; 2 Department of Neurology, Duke University, Durham, NC, United States of America; 3 Graduate School of Public Health, University of Pittsburgh, Pittsburgh, PA, United States of America; 4 Columbia University School of Social Work, New York, NY, United States of America; 5 The Michael J. Fox Foundation for Parkinson’s Research, New York, NY, United States of America; 6 Department of Neurology, University of Toronto, Toronto, Ontario, Canada; University of Rome Tor Vergata, ITALY

## Abstract

**Background:**

Fatigue is common in Parkinson’s Disease, but few effective treatments are available for it. Exploring triggers and alleviating factors, including effects of exercise, could inform development of management strategies for Parkinson’s Disease fatigue.

**Objectives:**

To examine triggers and alleviating factors for fatigue reported by individuals with Parkinson’s Disease, including perceived effect of exercise.

**Methods:**

A sample of individuals with self-reported Parkinson’s Disease participating in the study Fox Insight were administered an online survey. The survey included the Parkinson's Fatigue Scale, the Physical Activity Scale for the Elderly, and multiple-choice questions about triggers and alleviating factors for fatigue.

**Results:**

Among the sample of 1,029 individuals with Parkinson’s disease, mean (standard deviation (SD)) age was 67.4 (9.3) years, 44.0% were female. Parkinson’s Fatigue Scale score ranged from 16–80, mean (SD) 48.8 (16.2). Poor sleep (62.1%) and physical exertion (45.1%) were frequently reported triggers for fatigue. Coping strategies including sitting quietly (58.1%), laying down with or without napping, and exercise (20%). Physical Activity Scale for the Elderly scores were higher in those who reported that exercise alleviated their fatigue (49.7%) compared to those who reported it worsened their fatigue (18.9%) (mean (SD) score 158.5 (88.8) vs 119.8 (66.6) respectively; p<0.001).

**Conclusions:**

Several behavioral and environmental triggers and alleviating strategies for fatigue are reported by individuals with Parkinson’s disease. Many feel that exercise alleviates fatigue, though the relationship between exercise and fatigue in Parkinson’s Disease appears complex. This exploratory study may inform future development of treatments or coping strategies for Parkinson’s disease fatigue.

## Introduction

Fatigue in Parkinson’s disease (PD) occurs in over half of patients and has significant negative impact on both quality of life and the ability of patients to carry out daily activities [1–6]. Many patients find fatigue to be one of their most bothersome symptoms [7–9], yet few effective interventions for PD fatigue exist [10–12]. The etiology and pathophysiology of fatigue in PD is likely multifactorial [13–16], contributing to the challenges of developing therapeutics. While wearing off of PD medications may be associated with fatigue [17], and fatigue is levodopa-responsive in at least some patients [18, 19], other precipitating and alleviating factors for fatigue in PD are not well described. In particular, physical activity, which may be neuroprotective and is a core component of PD management [20–23], appears to have mixed effects on fatigue [24–27], but data are limited. To address this gap in knowledge, and toward informing future development of therapeutics, we examined patient-reported triggers and alleviating factors for fatigue in PD. Given the integral role exercise has in PD management and its potential as a treatment for PD fatigue [28–34], as well as the potential for physical activity to precipitate fatigue, we focused on patient-perceived effects of exercise on their fatigue.

## Materials and methods

### Sample

This work occurred as part of the Michael J Fox Foundation’s Parkinson’s Disease Education Consortium (PDEC) 2018 research program on the experience and impact of fatigue in PD, as described previously in detail [35]. Briefly, the sample was drawn from the Fox Insight (FI) cohort. FI is an online-only study that includes individuals aged 18 and older with self-reported Parkinson’s disease [36]. De-identified data used in this analysis are available on Fox Den at foxden.michaeljfox.org. Individuals with PD who were residents of the United States and had completed the Physical Activity Score for the Elderly (PASE) and Geriatric Depression Scale-15 item (GDS-15) in the prior 90 days (as part of their FI study assessments) received an email inviting them to participate in a survey about PD fatigue. Of the 3,531 FI participants who received the email invitation, 1,036 completed the survey (response rate 29.3%). Seven respondents were excluded for missing data (age, sex, year of diagnosis); thus, a final sample of 1,029 are included in this analysis.

This study was performed in accordance with the Declaration of Helsinki. This study and the FI study are approved by the New England Institutional Review Board (IRB; IRB study number for this study: 1279414, IRB study number for FI: 120160179), and online consent is obtained from each participant at enrollment.

### Assessments

Data collected as part of regularly-scheduled FI study assessments that were included in this analysis are:

- Demographics: age, sex, race
- PD disease duration (time from patient-reported month/year of diagnosis to month/year of survey participation)
- Movement Disorders Society Unified Parkinson’s Disease Rating Scale (MDS-UPDRS) part II, motor experiences of daily living [37]
- Geriatric Depression Scale-15 item (GDS-15) [38]
- Sleep and sleepiness were assessed with the Scale for Outcomes in Parkinson's disease-SLEEP [39]. This scale is recommended for assessing sleep in PD [39, 40]. It asks about pertinent symptoms in the prior month. It has a nighttime subscale (SCOPA-NS), maximum score 15, higher scores indicating worse subjective nighttime sleep; and daytime sleepiness subscale (SCOPA-DS), maximum score 18, higher scores indicating more daytime sleepiness.
- PD Medications: Participants were presented with a list of PD medications and could pick one or more. For purposes of this analysis, medications were grouped into 3 categories: levodopa, dopamine agonist, and other (including monoamine oxidase inhibitors and amantadine).
- Physical Activity Scale of the Elderly (PASE): The PASE is a self-reported measure of subjects’ recent activities, with higher PASE scores (range 0 to 500) indicative of higher levels of overall physical activity [41]. Subscores for light, moderate, strenuous and strength / endurance exercise per week were generated: frequency of exercise x duration per bout of exercise.

The survey related to fatigue that was administered to eligible FI participants included the following:

- The Parkinson Fatigue Scale (PFS) [42, 43], a PD-specific scale recommended for the evaluation of fatigue in PD [44]. A total score is generated as the sum of individual items, range 16 to 80. Higher scores are indicative of higher levels of fatigue. As described in scoring methods [42], subjects with a modified total score of at least 8 were categorized as “fatigued;” all others were categorized as “not fatigued.”
- A series of multiple-choice questions (MCQs) were developed based on results of a pilot phase that included telephone interviews with patients about their experience with PD fatigue, as previously described [35]. Each of these MCQs included an accompanying definition of fatigue, stating, ‘There are many ways in which to define fatigue. For the purpose of this survey, please think of fatigue as “an abnormal and excessive lack of energy".’ Table 1 includes the survey questions as administered to participants.

**Table 1:** Fatigue survey MCQs.

There are many ways in which to define fatigue. For the purpose of this survey, please think of fatigue as “an abnormal and excessive lack of energy".	
Question	Answer choices
Which of the following experiences, if any, can trigger or bring on fatigue for you? Please select all that apply.	[1] A poor night’s sleep
	[2] Physical exertion (e.g., from strenuous exercise or participating in sports)
	[3] Emotions, either negative or positive (such as stress, anger, joy, excitement, etc.)
	[4] Being overheated
	[5] Taking medication for Parkinson’s disease
	[6] A busy day
	[7] Some other trigger(s) (Those selecting “other” could then specify with a free text response)
	[8] Fatigue is not triggered by specific experiences for me
When you experience fatigue, what strategies do you most often use to cope with or recover from your fatigue? Please select up to three strategies.	[1] Sitting quietly (e.g., watching television, listening to the radio, reading, etc.)
	[2] Sleeping
	[3] Laying down to rest (not sleeping)
	[4] Exercising
	[5] Meditating
	[6] Sitting in a bath and/or a jacuzzi
	[7] Drinking coffee / caffeine
	[8] Taking medication
	[9] Eating
	[10] Some other coping strategy (Those selecting “other” could then specify with a free text response)
Respondents who selected [5] Taking medication, were presented with an additional question:	[1] Carbidopa-Levodopa (Sinemet, Sinemet CR, Sinemet Extended Release, Parcopa, Rytary, Stalevo, Duopa)
Which medication(s) do you most often take to cope with or recover from your fatigue? Please select all that apply.	[2] Entacapone (Comtan)
	[3] Pramipexole (Mirapex or Mirapex ER)
	[4] Ropinirole (Requip or Requip XL)
	[5] Rotigotine (Neupro patch)
	[6] Apomorphine (Apokyn)
	[7] Amatadine (Symmetrel or Gocovri)
	[8] Safinamide (Xadago)
	[9] Selegiline (Deprenyl, Eldepryl, Zelapar) or Rasagiline
	[10] Another medication (Please specify in the box below)
Many individuals with PD fluctuate between periods in which their symptoms are better controlled and periods during which symptoms return. We refer to the periods during which symptoms are better controlled as ON, and periods during which symptoms return as OFF. Based on this definition of OFF, do you experience OFF periods?	[1] Yes
	[2] No
Respondents who selected [1] Yes, were presented with an additional question:	[1] I am more likely to experience fatigue during OFF periods than during ON periods.
We refer to the periods during which symptoms are better controlled as ON, and periods during which symptoms return as OFF. Which of the following statements comes closest to your experience? Choose only one option.	[2] I am more likely to experience fatigue during ON periods than during OFF periods.
	[3] There is no relationship between fatigue and OFF for me—I am just as likely to experience fatigue during an OFF or ON period.
Which of the following statements comes closest to your view about exercise and fatigue? By exercise we mean any physical activity that lasts for at least 30 minutes, such as walking, playing basketball, dancing, bicycling, or gardening.	[1] Exercise makes me feel less fatigued physically, cognitively and emotionally.
	[2] Exercise makes me feel more fatigued physically, cognitively and emotionally.
	[3] Exercising doesn’t change the amount of fatigue I experience.
	[4] I do not exercise.

### Data analysis

Descriptive statistics were used to summarize demographics and questionnaire responses. In light of different manifestations of fatigue in males vs females with PD [5, 35, 45, 46], and in younger (<65 years) vs older (≥65 years) age groups [47–49], we compared these groups to explore whether there are also demographic differences in triggers and alleviating factors for fatigue, using chi^2^ test of homogeneity. Where the chi^2^ test omnibus p-value<0.05, adjusted residuals were compared post-hoc. A given demographic subgroup was considered significantly more likely to choose a given trigger or alleviating factor when the adjusted residual was >2.

To examine the relationship between exercise and fatigue, four groups were compared, defined according to their response on the question about perceived effect of exercise on fatigue (no effect, exercise improves fatigue, exercise worsens fatigue, or doesn’t exercise). Kruskal-Wallis was used to compare univariate differences in clinical characteristics and physical activity levels (PASE total score and subscores) among the four groups; based on Bonferroni adjustment, p<0.0125 was considered statistically significant.

Data were analyzed using Stata/IC 12.1 (StataCorp LP, College Station TX).

## Results

The final sample included 1,029 participants. Subject characteristics are shown in Table 2; mean (standard deviation) age was 67.4 (9.3) years, 56% were male, and mean disease duration was 4.6 years. There was a range of fatigue in the cohort; 465 (45.2%) of the sample were categorized as having fatigue based on dichotomized PFS score.

**Table 2:** Cohort characteristics.

	Total cohort N = 1029
**Sex**	
** Female**, n (%)	453 (44.0)
** Male**, n (%)	576 (56.0)
**Age in years**, Mean (SD)	67.4 (9.3)
**White or Caucasian,** n (%)	1002 (97.4)
**Disease duration in years**, Mean (SD)	4.6 (5.3)
**PD Medications**	
** Taking PD medications**, n (%)	908 (90.8)
** Levodopa**, n (%)	789 (76.7)
** Dopamine agonist**, n (%)	258 (25.1)
** Levodopa and dopamine agonist,** n (%)	185 (18.0)
** Other PD medication**, n (%)	337 (32.8)
**PFS score**, Mean (SD)	48.8 (16.2)
**MDS-UPDRS II score**, Mean (SD)	11.6 (8.0)
**GDS-15 score**, Mean (SD)	4.2 (3.7)
**SCOPA-NS,** Mean (SD)	6.14 (3.59)
**SCOPA-DS,** Mean (SD)	4.07 (3.27)
**PASE score**, Mean (SD)	141.6 (87.8)

### Triggers of fatigue

Self-reported triggers for fatigue are shown in Table 3.

**Table 3:** Triggers and coping strategies for fatigue reported by cohort as a whole and in demographic subgroups.

	n (% total cohort) n = 1029	Male, n (%) of total male n = 576	Female, n (%) of total female) n = 453	p-value (males vs females) *	Age < 65, n (%) of total age < 65) n = 344	Age ≥ 65, n (%) of total age ≥ 65) n = 685	p-value (age <65 vs age ≥65)*
**Patient-Reported Triggers**							
A poor night’s sleep	639 (62.1)	352 (61.1)	287 (63.4)	0.461	239 (69.5)	400 (58.4)	**0.001**
Physical exertion (e.g. strenuous exercise or participating in sports)	464 (45.1)	295 (51.2)	169 (37.3)	**<0.001**	160 (46.5)	304 (44.4)	0.517
A busy day	455 (44.2)	221 (38.4)	234 (51.7)	**<0.001**	186 (54.1)	269 (39.3)	**<0.001**
Emotions, either negative or positive (stress, anger, joy, excitement, etc.)	314 (30.5)	159 (27.6)	155 (34.1)	**0.022**	132 (38.4)	182 (26.6)	**<0.001**
Being overheated	228 (22.2)	119 (20.7)	109 (24.1)	0.192	91 (26.5)	137 (20.0)	**0.019**
Taking medication for PD	194 (18.9)	118 (20.5)	76 (16.8)	0.131	78 (22.7)	116 (16.9)	**0.026**
Fatigue is not triggered by specific experiences for me	128 (12.4)	71 (12.3)	57 (12.6)	0.902	30 (8.7)	98 (14.3)	**0.010**
Some other trigger(s)	78 (7.6)	35 (6.1)	43 (9.5)	**0.040**	27 (7.8)	51 (7.4)	0.818
**Patient-Reported Coping Strategies**							
Sitting quietly (e.g. watching TV, listening to radio, reading, etc.)	598 (58.1)	316 (54.9)	282 (62.3)	**0.017**	185 (53.8)	413 (60.3)	0.046
Laying down to rest (not sleeping)	541 (52.6)	286 (49.7)	255 (56.3)	**0.034**	195 (56.7)	346 (50.5)	0.061
Sleeping	495 (48.1)	300 (52.1)	195 (43.0)	**0.004**	176 (51.2)	319 (46.6)	0.164
Exercising	195 (19.0)	113 (19.6)	82 (18.1)	0.538	76 (22.1)	119 (17.4)	0.068
Drinking coffee / caffeine	169 (16.4)	99 (17.2)	70 (15.5)	0.456	79 (23.0)	90 (13.1)	**<0.001**
Taking medication	104 (10.1)	56 (9.7)	48 (10.6)	0.644	43 (12.5)	61 (8.9)	0.071
Eating	91 (8.8)	52 (9.0)	39 (8.6)	0.814	27 (7.8)	64 (9.3)	0.426
Meditating	80 (7.8)	35 (6.1)	45 (9.9)	**0.022**	28 (8.1)	52 (7.6)	0.757
Some other coping strategy	65 (6.3)	36 (6.3)	29 (6.4)	0.921	21 (6.1)	44 (6.4)	0.843
Sitting in a bath and/or jacuzzi	48 (4.7)	16 (2.8)	32 (7.1)	**0.001**	18 (5.2)	30 (4.4)	0.540

*bold values denote significant difference in proportion reporting specified trigger or alleviating factor based on post-hoc comparison of adjusted residuals if chi^2^-test omnibus p<0.05

The most frequently reported triggers were a poor night’s sleep (62.1%), physical exertion from exercise or sports (45.1%), and a busy day (44.2%). Other reported triggers included emotions (30.5%) and being overheated (22.2%). Twelve percent could not identify a specific trigger for fatigue. A trigger that was significantly more likely to be cited by males compared to females was physical exertion (51.2% vs 37.3%), whereas triggers more commonly cited by females compared to males included a busy day (51.7% vs 38.4%) and emotions (34.1% vs 27.6%). Respondents <65 were more likely to report a poor night’s sleep (69.5% vs 58.4%), a busy day (54.1% vs 39.3%), emotions (38.4% vs 26.6%), being overheated (26.5% vs 20.0%), and taking PD medication (22.7% vs 16.9%) as a trigger for fatigue.

Mean (SD) PFS score was 49.9 (15.8) among the 789 respondents taking levodopa, 50.0 (15.7) among the 258 respondents taking dopamine agonists, and 48.0 (16.4) among the 337 respondents taking other PD medications. PD medications were selected as a trigger for fatigue by 194 of respondents. Comparing these 194 participants to the 835 who did not select PD medications as a trigger for their fatigue, the proportion who reported taking an agonist was higher (n = 63 (33% of 194) versus n = 195 (23% of 835) respectively, p = 0.008), as was the proportion taking levodopa (n = 164 (85% of 194) versus n = 625 (75% of 835), p = 0.004), but there was no significant difference in the proportion taking “other” PD medications (n = 75 (39% of 194) versus n = 262 (31% of 835), p = 0.052). Additionally, among the 686 participants who reported experiencing OFF periods, 42.8% of those reported being more fatigued during medication OFF periods, 4.6% reported being more fatigued during medication ON periods, and 35.1% reported no relationship between fatigue and ON/OFF periods.

### Strategies for coping with fatigue

The most frequently reported coping strategies for fatigue (Table 3) were engaging in activities while sitting quietly (e.g. television or reading, 58.1%), laying down or resting without sleeping (52.6%), and sleeping (48.1%). A coping strategy that was more likely to be cited by males compared to females was sleeping (52.1% vs 43.0%), whereas coping strategies more commonly cited by females compared to males included sitting quietly doing an activity (62.3% vs 54.9%), laying down to rest without sleeping (56.3% vs 49.7%), meditating (9.9% vs 6.1%), and sitting in a bath or jacuzzi (7.1% vs 2.8%). Respondents <65 were more likely to employ drinking caffeine (23.0% vs 13.1%) as a coping strategy for fatigue. Of the 104 patients who reported that taking medications helped them cope with fatigue, 74 (71.2%) of those participants utilized formulations of levodopa, 7 (6.7%) utilized dopamine agonists (e.g. Pramipexole or Ropinirole), 5 (4.8%) utilized MAO-B inhibitors (Rasagiline or Selegiline), and 4 (3.8%) utilized benzodiazepines.

### Effect of exercise on fatigue

As shown in Table 3, 45.1% of participants reported physical exertion (e.g. from strenuous exercise or participating in sports) as a trigger for fatigue, while 19.0% reported using exercising as a coping strategy for fatigue. 51.2% of males reported physical exertion as a trigger for fatigue, compared to 37.3% of females (p<0.0001).

When queried specifically regarding whether they felt that exercise improved or worsened fatigue levels, approximately half of participants (49.7%) reported feeling that exercise improved fatigue (Table 4). A smaller proportion reported feeling that exercise either did not affect fatigue (18.6%) or worsened fatigue (18.9%), or that they do not exercise (9.5%). Of the 200 who reported that exercise improved their fatigue, 173 (86.5%) indicated exercise as a coping strategy for their fatigue. There were no significant differences in age, sex, or disease duration between groups.

**Table 4:** Characteristics and activity levels in relation to perceived effect of exercise on fatigue.

	Group 1 No effect of exercise	Group 2 Exercise improves fatigue	Group 3 Exercise worsens fatigue	Group 4: Does not exercise	p-value
**Clinical Characteristics**					
**n**^**1**^	191	511	194	98	—
**Age** in years, Mean (SD)	68.3 (10.0)	66.9 (9.0)	67.0 (9.3)	68.9 (9.6)	0.0552^2^
**Male** n (% of total male)	105 (18.2)	274 (47.6	126 (21.9)	52 (9.0)	0.048^3^
**Female** n (% of total female)	86 (19.0)	237 (52.3)	68 (15.0)	46 (10.2)	
**Disease duration** in years, Mean (SD)	5.1 (6.0)	4.2 (5.0)	4.6 (4.8)	5.6 (6.0)	0.1146^2^
**PFS score**, Mean (SD)	49.2 (16.5)	46.2 (14.9)	55.2 (14.9)	56.1 (15.2)	<0.0125^a,b,c,d,e^
**MDS-UPDRS II score**, Mean (SD)	12.4 (7.8)	9.8 (6.9)	14.1 (8.6)	16.3 (9.7)	<0.0125^a,c,d,e^
**GDS-15 score**, Mean (SD)	4.4 (3.7)	3.5 (3.2)	5.5 (4.0)	5.9 (4.2)	<0.0125^a,b,c,d,e^
**SCOPA-NS score, Mean (SD)**	6 (3.58)	5.90 (3.42)	6.80 (3.83)	7.11 (3.76)	<0.0125^d,e^
**SCOPA-DS score**, Mean (SD)	4.3 (3.5)	3.8 (3.0)	4.5 (3.6)	4.8 (3.4)	<0.0125^d,e^
**Physical Activity**					
**PASE total score**, Mean (SD)	144.8 (93.7)	158.5 (88.8)	119.8 (66.6)	85.2 (77.0)	<0.0125^a,c,d,e,f^
**Light sport score**, Median (IQR)	0 (0–2.3)	0 (0–2.3)	0 (0–2.3)	0 (0–0)	<0.0125^c,e,f^
**Moderate sport score**, Median (IQR)	0 (0–0.75)	0 (0–0.75)	0 (0–0)	0 (0–0)	<0.0125^c,e^
**Strenuous sport score**, Median (IQR)	0 (0–0.75)	0 (0–4.5)	0 (0–0)	0 (0–0)	<0.0125^a,c,d,e,f^
**Strength and Endurance exercises score**, Median (IQR)	0.75 (0–3.0)	1.8 (0–4.5)	0.75 (0–2.3)	0 (0–0)	<0.0125^c,d,e,f^

^**1**^35 participants did not provide a response to the question regarding impact of exercise on fatigue.

^2^p-value denotes any between-group differences per Kruskal-Wallis test. Significant differences (p <0.0125) among pairs of groups denoted as follows: (a) Group 1 vs Group 2 (b) Group 1 vs Group 3 (c) Group 1 vs Group 4 (d) Group 2 vs Group 3 (e) Group 2 vs Group 4 (f) Group 3 vs Group 4

^3^p-value as calculated with Fisher’s exact test

The group who reported that fatigue was improved by exercise had significantly lower MDS-UPDRS II score and GDS-15 score compared to all other groups (Table 4). They had significantly lower scores on SCOPA-DS compared to the groups who either felt that exercise worsened fatigue or did not exercise at all. PFS scores were lower in the group who felt that exercise helped their fatigue compared to those who felt that exercise worsened fatigue (mean PFS score 46.2 vs 55.2 respectively, p<0.001).

For the sample as a whole, PFS score and PASE total score were moderately inversely correlated (spearman’s ρ = -0.232, p<0.001). Those who felt that exercise helped their fatigue had significantly higher PASE scores compared to the other groups (Table 4). Of note however, light and moderate activity levels were not different between those who felt exercise improves versus worsens fatigue.

## Discussion

In this sample of individuals with PD with a range of fatigue severity, several triggers and alleviating factors for fatigue were identified. Physical activity emerged as both a trigger and alleviating factor for fatigue.

Several of the triggers for fatigue that were reported may be modifiable. Poor sleep was cited in this study as a major contributor to fatigue, especially among respondents younger than 65, highlighting the importance of assessing nighttime sleep in PD [50]. Additionally, a busy day was cited as a frequent triggering factor; this was reported more commonly by female participants and those younger than 65 years of age. As for other triggers, one-third of participants noted emotions as triggering fatigue; these participants were also more likely to be female and younger than 65. This warrants further study, as does the potential for including coping strategies for emotional distress, such as cognitive-behavioral therapy [51], in the therapeutic approach to PD fatigue. About one-fifth of participants cited over-heating as a trigger for their fatigue. Temperature dysregulation, including hypohidrosis, is seen in PD [52]. Counseling patients on the potential for high environmental temperatures to trigger fatigue may be warranted.

As for strategies to cope with fatigue, the most common ones were not surprising: resting, with or without sleep. Our study suggests than males tend to prefer sleeping, whereas females tend to prefer resting without sleeping. These findings may reflect sex differences in napping propensity and the relationship between nighttime sleep, daytime sleepiness, and fatigue [53, 54]. Increasing data support the utility of daytime napping in PD [55], but the timing and duration of daytime naps needs to be balanced with potential effects on nighttime sleep. Caffeine is used to manage fatigue by a substantial minority in our sample, especially respondents younger than 65 years of age. This raises the possibility that caffeine may not only help daytime sleepiness but also fatigue in PD [56], and warrants further study.

Our results highlight a complex relationship between dopaminergic medications and fatigue in PD. Fatigue scores were similar among patients taking different types of PD medications (levodopa vs dopamine agonists vs other PD meds). Both intake of PD medications and wearing off of those medications emerged as a subjective trigger for fatigue. The proportion taking levodopa and dopamine agonist was higher among those who reported PD medication as a trigger for fatigue. These results must be interpreted with caution; whether these findings reflect medication-induced fatigue or whether disease severity or other patient characteristics confound the relationship between fatigue and medications requires further study. Nevertheless, sleepiness resulting from dopaminergic medications, especially dopamine agonists but also levodopa, is well documented [57]. Some patients may experience this as a sense of fatigue as well, or may not necessarily make a distinction between sleepiness and fatigue [35]. In a patient with PD fatigue, especially one being treated with a dopamine agonist, consideration for medication-induced hypersomnolence is important. A careful clinical history may help distinguish between hypersomnolence and fatigue. In some patients who cannot make this distinction, an adjustment of dopaminergic medication may still be considered. In addition, our findings reinforce the importance of assessing for non-motor symptoms such as fatigue as a manifestation of OFF periods [58, 59], and adjusting PD medications accordingly.

Half of participants reported that exercise improved fatigue levels and fatigue severity was lower in that group. Additionally, roughly one-fifth of participants reported using exercise as a coping strategy for fatigue; this did not vary significantly by sex or age. Our study design does not allow for examination of causality, but data from other patient populations indicates that exercise could have a potential benefit on fatigue. Exercise improves cancer-related fatigue [60] and chronic fatigue syndrome [61]. The potential utility of exercise to improve PD fatigue is important to explore further.

By contrast, nearly one in five participants reported that exercise worsens their fatigue. Total PASE scores were lower in the group that reported worsening of fatigue with exercise (though light/moderate exercise/activity levels were similar to those who feel that exercise improves their fatigue). Fatigue has been identified as a barrier to exercise in PD patients with fatigue [62], as has low outcome expectations, i.e. lack of expectation for exercise to derive benefit [63]. These factors could explain the lower levels of physical activity in the group who reported exercise worsens their fatigue. Fatigue occurring as a result of physical exercise is not unique to the PD population and has been reported by other disease populations [64–66]. In chronic fatigue syndrome, Black et al suggest that vigorous exercise or a 30% increase in activity can trigger a relapse [65]. On the other hand, those individuals who reported that exercise exacerbates their fatigue had more severe disease and it is likely that other factors besides fatigue contributed to low physical activity levels. For example, participants who were more depressed also tended to exercise less and to be less active. Daytime sleepiness is also an important non-motor symptom that can be difficult for patients to distinguish from fatigue; in our respondents, those with both worse subjective nighttime sleep and greater daytime sleepiness also tended to feel less positively about the effects of exercise on fatigue. A further exploration of how other non-motor comorbidities may influence activity levels and perceived effects of exercise on PD fatigue, including not only depression [24] and sleep disturbances [50, 67], but also apathy and dysautonomia [68], is needed.

Another key aspect to understand in the relationship between exercise and fatigue is the influence of intensity of exercise. Physical exertion at an intensity or duration which exceeds the individual’s capabilities can exacerbate fatigue [65, 69]. While high intensity exercise may offer therapeutic benefits on motor function and progression beyond low-intensity exercise, [30, 70–73], the effects of high intensity exercise on PD fatigue are less understood. It is likely that there are subgroups of PD patients that vary according to perceived and physiological effects of exercise on their motor and non-motor symptoms and especially fatigue, and understanding the determinants of this is critical. This in turn can inform individualized exercise prescriptions, which account for patient characteristics, comorbidities, fatigue severity, and other non-motor symptoms, in order to maximize the benefits of exercise in PD while avoiding exacerbating fatigue, or even potentially helping it.

There are several limitations to our study. Our study lacks a healthy control group, and conclusions cannot be drawn regarding whether the experiences of fatigue captured in our sample of PD patients is significantly similar or different from fatigue experienced by the general non-PD population. Because the study was conducted via online survey, we were only able to capture data from PD patients with the technological and socioeconomic ability to access the internet. The respondents on Fox Insight were predominantly white [36], and results may not be broadly applicable to all racial groups. Our survey response rate of 29.3% also limits generalizability. The PASE is self-administered in our study, as opposed to being conducted via interview with a trained administrator; however, in other instances where PASE was self-administered, subjects were as likely to over-categorize as under-categorize their activity level, and the magnitude of miscategorization was small [74]. The survey did not query the dosages of medications that respondents are taking, limiting our ability to examine the dose-dependent relationships between fatigue and dopaminergic medication. Similarly, the survey did not query the specific types of exercises that respondents are employing as coping strategies for fatigue. Both of these domains would be valuable to investigate in future research. And finally, due to the observational, cross-sectional nature of the study, we are unable to draw conclusions regarding causality of associations.

## Conclusion

Our study describes some of the self-reported factors that affect fatigue in PD patients. Many different factors were reported by patients to worsen fatigue, including physical activity, poor sleep, and medication wearing off. Exercise was also reported to help fatigue in half of patients but was perceived to worsen fatigue in others. From a practical standpoint, our results demonstrate that some patients can often identify triggers for their fatigue; counseling patients to keep symptom diaries or otherwise explore their individual habits may in turn allow them to avoid triggers or to institute interventions. Similarly, patients use a variety of coping mechanisms for their fatigue. Our results suggest several potential strategies that require further investigation, including strategic daytime napping, the use of caffeine, exercise, and strategies to cope with emotions. Demographic differences in triggers and coping strategies indicate that a personalized approach may be important, and clinical trials investigating treatments for PD fatigue may benefit from stratifying by age and sex when examining treatment effects. The perceived effect of exercise was more mixed; additional study is needed to further elucidate the effects of exercise upon fatigue in PD patients, and especially to determine how exercise programs need to be tailored for PD subgroups in relation to their fatigue.
